# scCompass: An Integrated Multi‐Species scRNA‐seq Database for AI‐Ready

**DOI:** 10.1002/advs.202500870

**Published:** 2025-05-02

**Authors:** Pengfei Wang, Wenhao Liu, Jiajia Wang, Yana Liu, Pengjiang Li, Ping Xu, Wentao Cui, Ran Zhang, Qingqing Long, Zhilong Hu, Chen Fang, Jingxi Dong, Chunyang Zhang, Yan Chen, Chengrui Wang, Guole Liu, Hanyu Xie, Yiyang Zhang, Meng Xiao, Shubai Chen, Haiping Jiang, The X‐Compass Consortium, Yiqiang Chen, Ge Yang, Shihua Zhang, Zhen Meng, Xuezhi Wang, Guihai Feng, Xin Li, Yuanchun Zhou

**Affiliations:** ^1^ Computer Network Information Center Chinese Academy of Sciences Beijing 100083 China; ^2^ State Key Laboratory of Stem Cell and Reproductive Biology Institute of Zoology Chinese Academy of Sciences Beijing 100101 China; ^3^ Institute for Stem Cell and Regenerative Medicine Chinese Academy of Sciences Beijing 100101 China; ^4^ Beijing Institute for Stem Cell and Regenerative Medicine Beijing 100101 China; ^5^ State Key Laboratory of Multimodal Artificial Intelligence Systems Institute of Automation Chinese Academy of Sciences Beijing 100190 China; ^6^ CEMS NCMIS HCMS MDIS RCSDS Academy of Mathematics and Systems Science Chinese Academy of Sciences Beijing 100190 China; ^7^ Beijing Key Laboratory of Mobile Computing and Pervasive Device Institute of Computing Technology Chinese Academy of Sciences Beijing 100190 China; ^8^ University of Chinese Academy of Sciences Beijing 100864 China; ^9^ College of Life Science Northeast Agricultural University Harbin 150030 China

**Keywords:** AI‐ready, multi‐species, scRNA‐seq database, single‐cell

## Abstract

Emerging single‐cell sequencing technology has generated large amounts of data, allowing analysis of cellular dynamics and gene regulation at the single‐cell resolution. Advances in artificial intelligence enhance life sciences research by delivering critical insights and optimizing data analysis processes. However, inconsistent data processing quality and standards remain to be a major challenge. Here scCompass is proposed, which provides a comprehensive resource designed to build large‐scale, multi‐species, and model‐friendly single‐cell data collection. By applying standardized data pre‐processing, scCompass integrates and curates transcriptomic data from nearly 105 million single cells across 13 species. Using this extensive dataset, it is able to identify stable expression genes (SEGs) and organ‐specific expression genes (OSGs) in humans and mice. Different scalable datasets are provided that can be easily adapted for AI model training and the pretrained checkpoints with state‐of‐the‐art single‐cell foundation models. In summary, scCompass is highly efficient and scalable database for AI‐ready, which combined with user‐friendly data sharing, visualization, and online analysis, greatly simplifies data access and exploitation for researchers in single‐cell biology (http://www.bdbe.cn/kun).

## Graphical abstract

scCompass integrates and standardizes transcriptomic data from ≈105 million single cells across 13 species, enabling the identification of stable expression genes and organ‐specific expression genes in humans and mice. It provides scalable datasets for AI model training, pretrained model checkpoints, and user‐friendly platforms for data sharing, visualization, and analysis, facilitating advanced single‐cell research and AI applications.

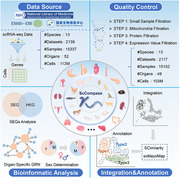

## 1 Introduction

Cells are the fundamental units of life and a central focus of life sciences research.^[^ ^1^, ^2^, ^3^ ^]^ High‐throughput RNA sequencing enables to capture RNA in a large scale to reveal the whole genome‐expression profiling.^[^ ^4^, ^5^ ^]^ The emergence of single‐cell RNA sequencing (scRNA‐seq) can uncover the genetic structure and gene expression states of individual cells, reflecting cellular heterogeneity. The application of scRNA‐seq has facilitated the generation and accumulation of vast amounts of single‐cell omics data. This enables further insight into exploration of life processes and to understand cellular heterogeneity.^[^ ^6^, ^7^ ^]^

Extensive single‐cell datasets are essential for uncovering fundamental biological processes, transcriptomic foundation models offer potential to reveal complex interactions and regulatory mechanisms within organisms. The training of foundation models requires a large amount of high‐quality transcriptomic datasets, like GeneCompass^[^ ^8^ ^]^ (100 million cells), scGPT^[^ ^9^ ^]^ (80 million cells), and Geneformer^[^ ^10^ ^]^ (30 million cells). Their experimental results demonstrate that as data volumes increase, model performance correspondingly improves, emphasizing the urgent need for large, standardized, and model‐friendly datasets. But the data integration and standardization pose great challenges. Platforms like CELLxGENE,^[^ ^11^ ^]^ Human Cell Landscape (HCL),^[^ ^12^ ^]^ and Tabula Muris^[^ ^13^ ^]^ have improved the usability and integration of multiple datasets. CELLxGENE explores data to understand the mechanisms of human health. HCL is specialized in human gene expression, whereas Tabula Muris focuses on mouse single cells. While these developments are promising, significant challenges still remain in terms of data consistency and accessibility. In particular, existing databases suffer from limitations such as limited organisms, a lack of standardized procedures, inefficiencies, and inaccuracies at downstream annotation processes, or inadequate quality control.

Therefore, we provide a single‐cell dataset, scCompass, which provides a consistent process spanning humans, mice, and other 11 species. We first gathered over 2 petabytes (PB) of raw single‐cell RNA sequencing (scRNA‐seq) data from public resources. Next by employing a unified data analysis and quality control (QC) process, we constructed detailed single‐cell atlases for each species. These datasets consist of nearly 105 million single‐cell transcriptomes. Leveraging this large‐scale single‐cell dataset, we aim to analyze and extract biological insights that are difficult to uncover in smaller datasets.^[^ ^14^, ^15^ ^]^ Traditional housekeeping genes (HKGs) have been identified through large‐scale bulk gene expression analysis.^[^ ^16^, ^17^ ^]^ In this study, we leveraged single‐cell data to explore and identify a novel set of stable expression genes (SEGs) at the single‐cell level. These genes offer a promising foundation for future reference gene evaluation in transcriptomic research. scCompass includes over 30 organs (organs: exclude immune cells and nervous system) from humans and mice. We identify genes with organ‐specific expression genes (OSGs) according to metadata, offering a valuable resource for elucidating the functional characteristics of different organs.

As the need for AI‐ready single‐cell datasets is growing, providing high‐dimensional data with rich cellular detail has the potential to foundation model pretraining.^[^ ^18^ ^]^ To maximize this potential, we have constructed a series of datasets across varying scales, each carefully prepared through median normalization, expression value ranking, and standardized processing of filtered single‐cell expression matrices. These datasets are optimized for direct integration with SOTA single‐cell foundation models, including GeneCompass,^[^ ^8^ ^]^ scGPT,^[^ ^9^ ^]^ and Geneformer,^[^ ^10^ ^]^ and the pretrained foundation models are provided to enable researchers diverse single‐cell downstream tasks. Furthermore, the standardized dataset can also be utilized for fine‐tuning. By developing high‐quality, AI‐ready datasets, we aim to provide a sound basis for optimizing dataset construction, reducing inter‐dataset heterogeneity, accelerating AI model iteration, enhancing model generalization, and supporting AI‐driven life sciences research.

We developed scCompass, a high‐quality, uniformly processed dataset, from which we identified SEGs and OSGs. This resource provides AI‐ready training datasets of various scales tailored to different foundational models, along with pretrained models. Additionally, scCompass features an interactive, user‐friendly platform for data retrieval and sharing, enhancing accessibility for research and application.

## 2 Results

### 2.1 Construction of scCompass, a Unified, Large‐Scale, Multi‐Species Single Cell Transcriptomic Data

**Figure 1:**
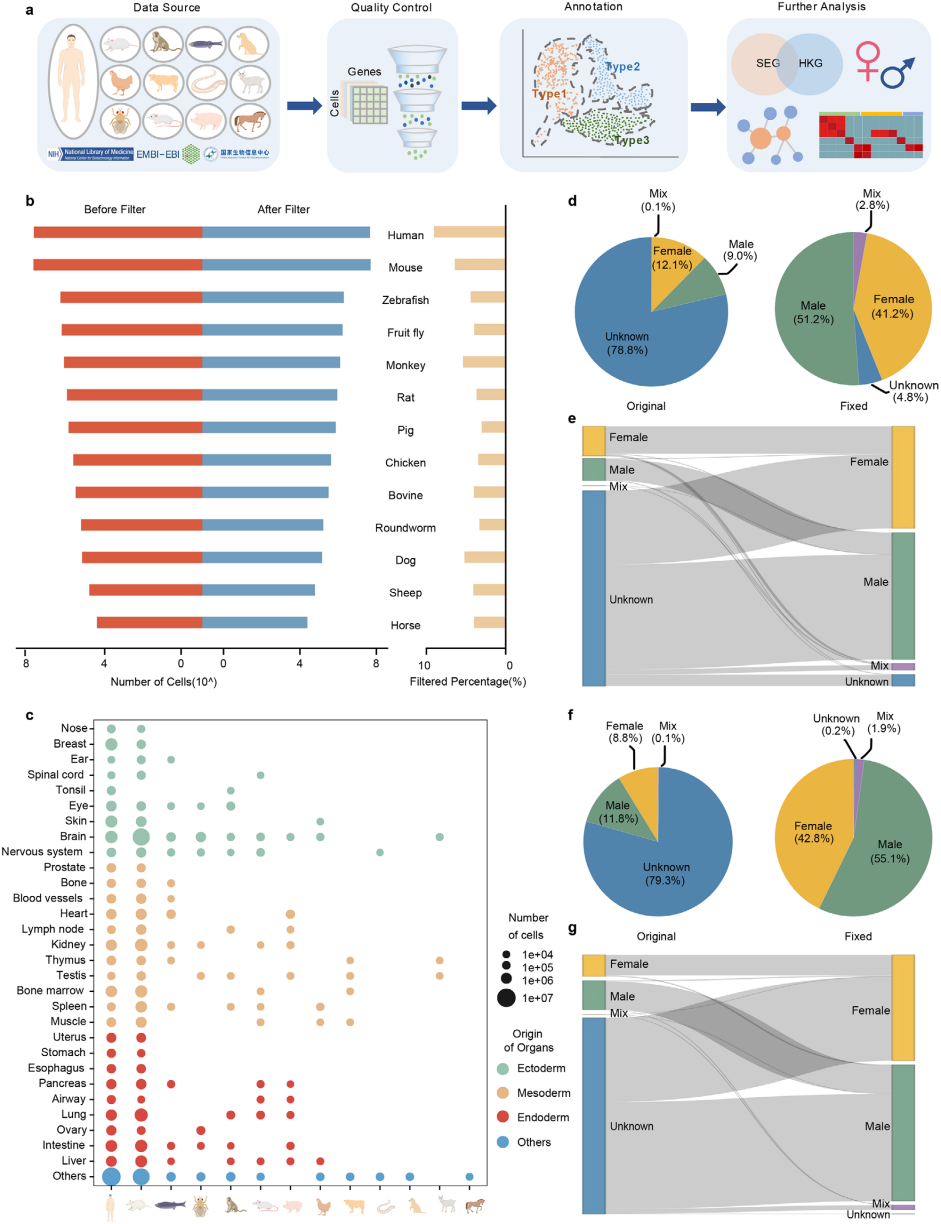
Data curation pipeline of scCompass. a) Illustration of scCompass data collection, QC, cell type annotation, and downstream analysis. b) Cell counts for 13 species before and after QC (left panel), along with the percentage of filtered‐out cells (right panel). c) Bubble chart depicting the cell proportions and developmental origins of organs across 13 species. d–g) Sex correction in humans and mice. d) Human sex distribution before (left panel) and after correction (right panel). e) The Sankey diagram illustrates the results of sex correction for each human sample: left, original label, right, corrected label. f) Mouse sex distribution before (left panel) and after correction (right panel). g) The Sankey diagram illustrates the results of sex correction for each mouse sample: left, original label, right, corrected label.

Using a structured curation model (**Figure** **1**a), we integrated a multi‐species (spanning human, mouse, and other 11 species) single‐cell transcriptome datasets from public repositories NCBI GEO,^[^ ^19^ ^]^ EMBL‐EBI ArrayExpress,^[^ ^20^ ^]^ and CNCB.^[^ ^21^ ^]^ We first downloaded and analyzed all raw data that passed our screening, that is ≈113 million expression profiles derived from a total of 15 337 samples, preprocessed using the CellRanger with default parameters. After QC, 104 637 783 cells remain, effectively filtering out less than 10% of cells in each species. Specifically, there are ≈50 million cells each for human and mouse. (Figure 1b; Figure S1a, Supporting Information). By observing the distribution of cell numbers across organs (Figure 1c), we found that humans and mice have the most comprehensive coverage of organ types. This allows us to perform further analysis, including those of the OSGs.

We then meticulously annotated each dataset with species, sex, tissue, and cancer/normal distinction (Figure 1d,f; Figure S1b, Supporting Information). We observed that the sex information for a majority of human and mouse samples was labeled as “unknown”, with 78.8% of human samples and 79.3% of mouse samples lacking explicit sex designation. To address this, we defined X‐Ratio and Y‐Ratio thresholds (see Experimental Section) by examining at the true distribution of X and Y chromosome gene expression in sex‐specific tissues such as testis and ovary (Figure S1c–f, Supporting Information). Using these thresholds, we were able to assign sex attributes to the majority of samples previously labeled as “unknown” and verify the accuracy of existing sex labels. As a result, the proportion of samples with “unknown” sex attributes significantly decreased to 7.3% in the human dataset and 2.1% in the mouse dataset.

Overall, we construct a standardized and unified multi‐species dataset, scCompass, following a structured curation process and provide a sex correction method.

### 2.2 Constructing Single‐Cell Atlas of scCompass

Cell type annotation is essential to understand cellular heterogeneity. Our curated large‐scale datasets display considerable variability in both annotation methods and quality. Therefore, we implemented a consistent cell type annotation process and created basic landscape of scCompass.

**Figure 2:**
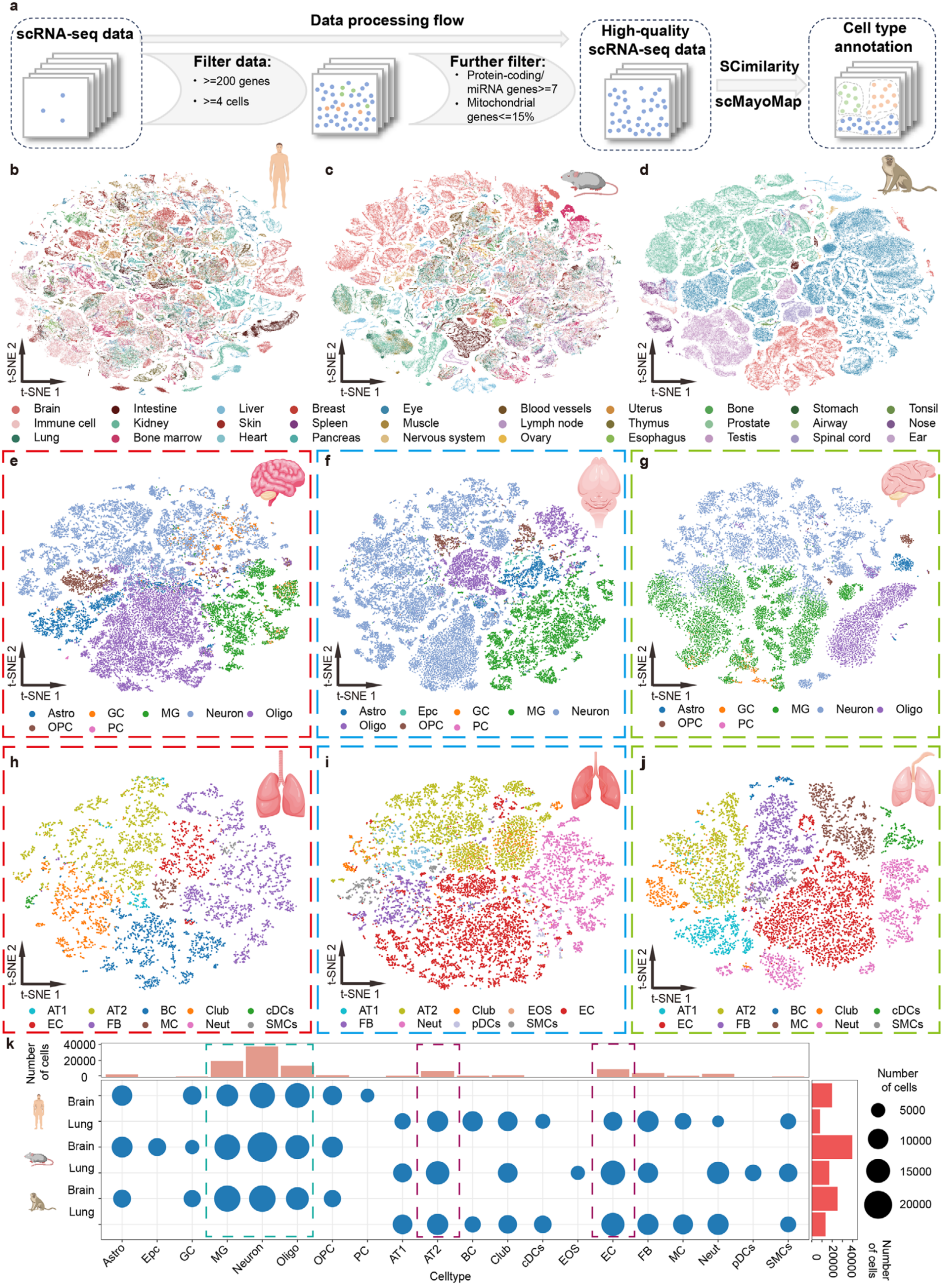
Single‐cell atlas construction of human, mouse, and monkey. a) Cell QC and cell type annotation (SCimilarity and scMayoMap). b–d) Single‐cell atlas of human, mouse, and monkey with t‐SNE, colored dots represent different tissues. e–g) Cell types in the brain of humans e), mouse f), and monkey g), colored dots represent different cell types. h–j) Cell types in the lung of human h), mouse i), and monkey j), colored dots represent different cell types. k) Statistics of the proportion and quantity of cell types in the brain and lung for humans, mice, and monkeys. Astro, Astrocyte. Epc, Ependymal Cell. GC, Glial Cell. MG, Microglial Cell. Oligo, Oligodendrocyte. OPC, Oligodendrocyte Precursor Cell. PC, Pericyte. AT1, Type I Pneumocyte. AT2, Type II Pneumocyte. BC, Basal Cell. cDCs, Conventional Dendritic Cell. EOS, Eosinophil. EC, Endothelial Cell. FB, Fibroblast. MC, Mast Cell. Neut, Neutrophil. pDCs, Plasmacytoid Dendritic Cell. SMCs, Smooth Muscle Cell.

First, we performed a QC process on scCompass to only include high‐quality cells. Initially, cells with fewer than 200 detected genes or samples with fewer than 4 cells were excluded. Subsequently, we removed cells expressing less than 7 protein‐coding genes and mitochondrial gene expression accounted for over 15% in total. Due to the large volume of scCompass, it is impractical to label the cells with traditional manual annotation methods, we utilized cell type annotation tools SCimilarity^[^ ^22^ ^]^ and scMayoMap,^[^ ^23^ ^]^ total more than 200 cell types were annotated, covering the majority cell types (**Figure** **2**a). As shown in Figure S2a (Supporting Information), we evaluated the annotation accuracy of scMayoMap. The results demonstrated that both scMayoMap and SCimilarity achieved an average annotation accuracy of over 80%. To further validate the applicability of these tools in multi‐species datasets, as shown in Figure S2b (Supporting Information), we tested scMayoMap and SCimilarity on datasets from different species. The results indicated that SCimilarity outperformed scMayoMap in multi‐species annotation accuracy. Therefore, we selected SCimilarity as the annotation tool for multi‐species datasets to ensure high‐precision cell type annotation across different species.

Then we illustrate the atlas of randomly selected single cells (297 403 human cells, 297 324 mouse cells, and 292 131 monkey cells) across the top 30 tissues (Figure 2b–d, for other species Figure S3, Supporting Information). We focused on the brain and lung, the top two organs in terms of cell numbers. We present the cell types identified in the brain tissues of humans, mice, and monkeys, with cell counts of 37 423, 39 368, and 25166, respectively. The main cell types observed include oligodendrocytes, oligodendrocyte precursor cells, microglial cells (MGs), astrocytes, glutamatergic neurons, neural cells, etc. (Figure 2e–g). Similarly, we display the lung tissue cell distributions for humans, mice, and monkeys, comprising 7385, 16887, and 13025 cells, respectively. The major cell types in lung tissues were identified as alveolar type I (AT1) cells, alveolar type II (AT2) cells, and endothelial cells, etc. (Figure 2h–j). The annotation results indicate that the major cell types for each organ are well‐represented.

Finally, we illustrate the hierarchical clustering of different cell types in the brain and lung (Figure 2k). In the brain, neurons, MG, and oligo are abundant, displaying similar distribution among species, highlighting their crucial roles in neural function and maintenance (cyan dashed box). Likewise, in the lung, AT2 and endothelial cell (EC) were predominant, with consistent proportions across species (purple dashed box). These findings highlight the presence of key functional cell types in the brain and lung and underscore the multi‐species conservation of those cell types.

Overall, we construct the single‐cell atlas for scCompass. Our cell type annotation covers all major cell types in species.

### 2.3 Identification of SEGs from Large Datasets

Unlike traditional bulk RNA sequencing data, scRNA‐seq provides insights into significant transcriptional differences at the individual cell level.^[^ ^24^, ^25^ ^]^ On the cell population level, a subset of genes known as HKGs consistently display stable expression across various tissues.^[^ ^17^, ^26^ ^]^ Therefore, identifying SEGs within large‐scale single‐cell data is essential for basic cellular function analysis.

**Figure 3:**
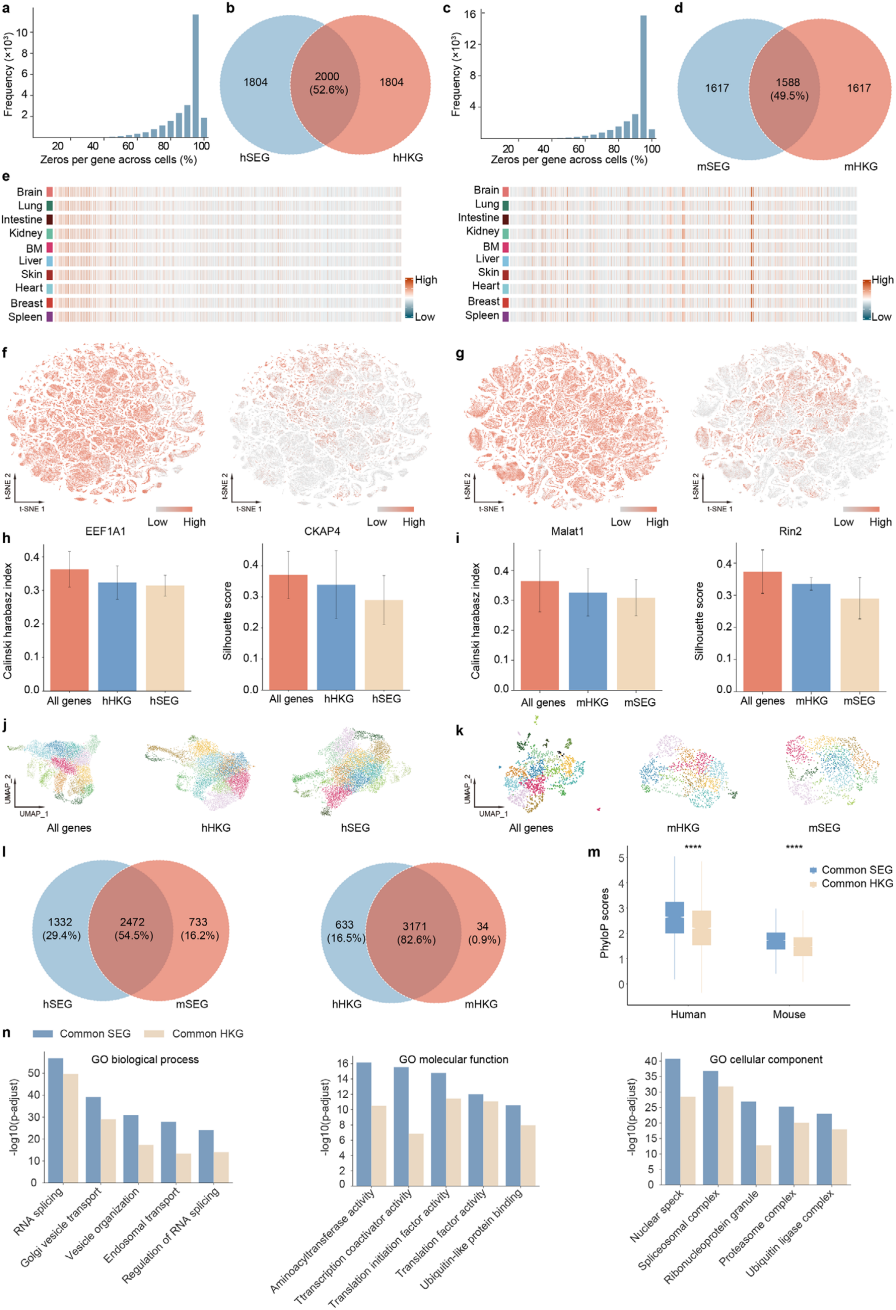
SEG analysis of human and mouse. a) The zeros per gene across human single‐cell data. b) Number of common genes between SEGs and HKGs for humans with venn plot. c) The zeros per gene across mouse data. d) Number of shared genes between SEGs and HKGs for a mouse. e) Heatmap showing the distribution of SEGs in the top 10 organs for humans (left panel) and mice (right panel). f) tSNE visualization showing the expression pattern of EEF1A1 of unique hSEGs and CKAP4 of unique hSEGs, respectively, in indicated the top 30 organs of humans. g) tSNE visualization showing the expression pattern of Malat1 of unique mSEGs and Rin2 of unique mSEGs, respectively, in indicated the top 30 organs of the mouse. h,i) K‐means clustering of 20 randomly sampled single‐cell data evaluated with the Calinski‐Harabasz index and silhouette score, using all expressed genes, HKGs, and SEGs identified from this study for human (hSEGs) and mouse (mSEGs). j,k) UMAP plots generated from human j) and mouse k) single‐cell data randomly selected from 20 samples using all expressed genes, HKGs, or SEGs. l) Common SEGs and HKGs between humans and mice. m) Comparison of conservation for common SEGs and HKGs in human and mouse genomes, with *p* values calculated using a two‐sided Wilcoxon rank‐sum test. n) Overrepresentation analysis of SEGs shared between hSEG and mSEG (common SEGs) and HKGs shared between hHKG and mHKG (common HKG), using Gene Ontology (GO). (BM, Bone Marrow).

Gene expression patterns differ across tissues, we quantified the distribution of zeros per gene at the single‐cell level in humans and mice. Most genes exhibit zero expression in 80% of cells, indicating that ranking by zero‐value rates effectively reflects gene stability across different cells (Figure 3a,c). Then, we selected the same number of ranked genes according to the human and mouse HKG lists, named SEGs (Figure 3b,d). We found that SEGs and HKGs share 52.6% overlap in humans and 49.5% in mice, suggesting intersection but also distinctiveness of SEGs.

To demonstrate stable expression across different organs, we investigated SEG and HKG expression levels in pseudo‐bulk data across the top 10 organs for both humans and mice, they both displayed stable expression (Figure 3e; Figure S4a,b, Supporting Information). The unique SEGs expression pattern is more stable than HKGs at single‐cell resolution. For example, unique SEGs (EEF1A1, Malat1) showed pan‐expression pattern while HKGs (CKAP4, Rin2) showed imbalanced expression in top 30 tissues (Figure 3f,g; Figure S4c,d, Supporting Information).

To further validate the stability of SEGs, we evaluate the performance of all genes, HKGs, and SEGs at clustering using Calinski harabasz index^[^ ^27^ ^]^ and Sihouette score.^[^ ^28^ ^]^ The results showed that clustering with all genes performed best, followed by HKGs, with SEGs yielding the lowest performance (Figure 3h,i). The UMAP visualizations (Figure 3j,k) illustrate that cell clusters become less distinct from all genes to SEGs. This finding suggests that while SEGs are more stable, they contribute less to the variance needed for clustering since clustering algorithms rely on genes with variable expression to distinguish cell types.

Then we analyzed the evolutionary conservation of common SEGs (genes overlap between hSEGs and mSEGs) and common HKGs (genes overlap between hSEGs and mSEGs). The phyloP scores reveal that common SEGs exhibit significantly higher conservation (Figure 3m). This suggests that SEGs may play fundamental roles during evolution. Subsequent Gene Ontology (GO) enrichment analysis (Figure 3n) demonstrated that common SEGs are more enriched across GO terms compared to commonly defined HKG.

Finally, We visualized the expression patterns with UMAP of EEF1A1 and HSP90AA1 (Figure S5a,b, Supporting Information) in human time‐series single‐cell^[^ ^29^ ^]^ and Malat1 and Ddx5 (Figure S5c,d, Supporting Information) in mouse time‐series single‐cell.^[^ ^30^ ^]^ We found that these genes exhibit high expression stability across different developmental stages in both humans and mice, suggesting that they may play fundamental roles during evolution by maintaining essential cellular functions and regulatory mechanisms over time. This further validates the SEGs reliability at single‐cell resolution and highlights the potential importance in evolutionary processes. With the high‐resolution scCompass dataset, we identified SEGs at the single‐cell level that exhibit exceptional stability and pronounced evolutionary conservation.

### 2.4 Definition of Organ‐Specific Expression Genes

**Figure 4:**
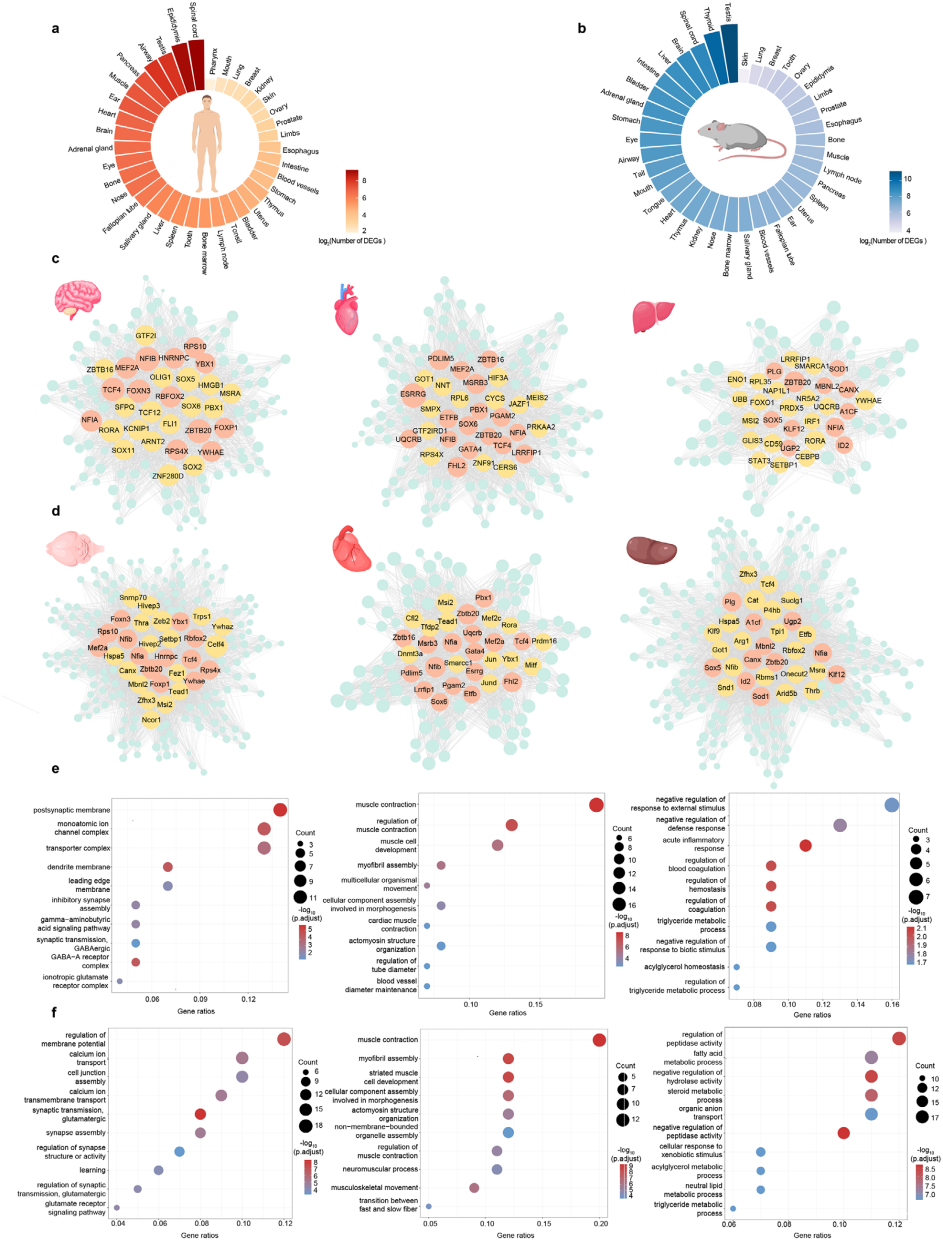
Organ differential expression gene analysis of human and mouse. a,b) Log values of differential expression genes in various organs between human and mouse. c,d) GRN constructed with OSGs of brain (left panel), heart (middle panel), and liver (right panel). Nodes with orange color represent the common TFs between human and mouse, the yellow nodes represent the distinct TFs of human or mouse, and the blue nodes represent the OSGs. e,f) GO enrichment related to cellular functions of predicted target genes in brain, heart, and lung. Dot color represents significance level of enrichment analysis and dot size is count of target genes classified in GO terms. *p* values were calculated using a hypergeometric test. Multiple comparisons adjustment was performed using the Benjamini and Hochberg method.

OSGs offer key insights into the specialized functions unique to each organ. The scCompass dataset covers 37 diverse organs, enabling a comprehensive and systematic identification of OSGs across a broad spectrum of organs (**Figure** **4**a,b).

To explore the regulatory mechanisms underlying these OSGs, we constructed organ‐specific GRNs of three vital functional organs (brain, heart, and liver) (Figure 4c,d). Specifically, we identified 13 common TFs in the brain, 17 in the heart, and 11 in the liver. Several regulators for human brain (NFIB^[^ ^31^ ^]^ and OLIG1^[^ ^32^ ^]^), human heart (PDLIM5^[^ ^33^ ^]^ and FHL2^[^ ^34^ ^]^), human liver (FOXO1^[^ ^35^ ^]^ and ID2^[^ ^36^ ^]^), mouse brain (Msi2^[^ ^37^ ^]^ and Nfib^[^ ^38^ ^]^), mouse heart (Pdlim5^[^ ^39^ ^]^ and Tead1^[^ ^40^ ^]^), and mouse liver (Id2^[^ ^41^ ^]^ and Onecut2^[^ ^42^ ^]^), have been reported in the literature that is critically important in organs.

To elucidate the biological functions of these OSGs, we conducted GO analysis for the three organs (Figure 4e,f). The enriched functions corresponded closely with the physiological roles of each organ. Specifically, in the human brain, OSGs were enriched in functions such as postsynaptic membrane and monoatomic ion channel complex, and in the mouse brain, they were associated with regulation of membrane potential and calcium ion transport. The muscle contraction is enriched both in the human and mouse heart. The human liver's OSGs were involved in regulation of blood coagulation and triglyceride metabolic processes, while in the mouse liver, they were enriched in fatty acid metabolic processes and steroid metabolic processes.

Using the comprehensive organ metadata from scCompass, we analyzed the gene expression matrix to systematically identify OSGs for each organ.

### 2.5 Evaluation of scCompass for AI‐Ready Applications

**Figure 5:**
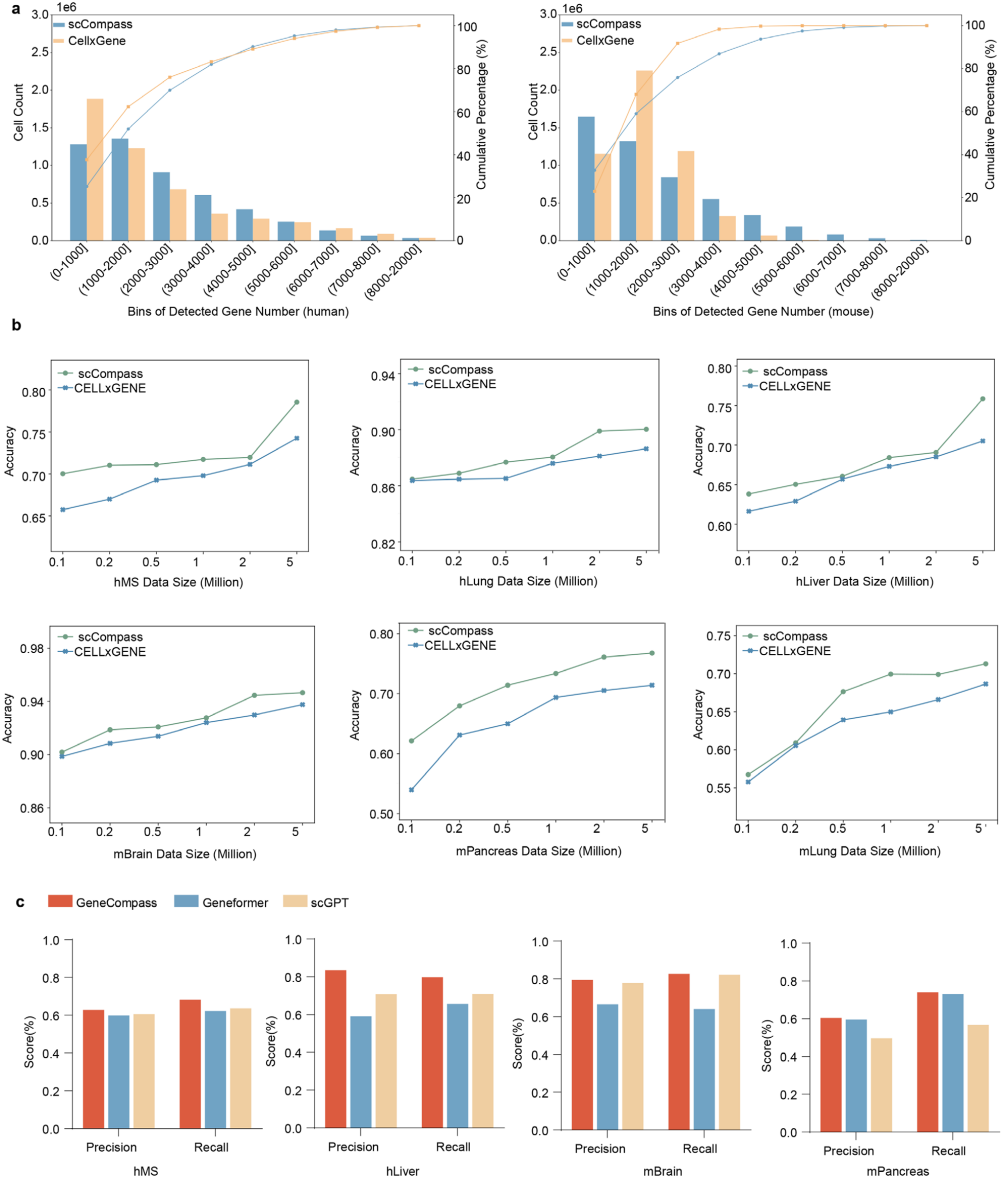
Evaluation of scCompass AI‐Ready adaptability. a) A Pareto chart illustrates the gene expression profiles of cells sampled from scCompass and CELLxGENE. b) Accuracy of GeneCompass models trained on various scales of single‐cell samples, evaluated on hMS, hLung, hLiver, mBrain, mPancreas, and mLung for cell type annotation. In the line plots, the green dots line represents pretraining with scCompass data, while blue dots line represents pretraining with CELLxGENE data. c) Precision and recall evaluation of cell type annotation tasks for different models (scGPT, Geneformer, GeneCompass) on 5 million human sampled cells.

Current single‐cell transcriptomic foundation models require different input data formats: scGPT uses only single‐cell expression matrices, Geneformer relies on ranked gene expression sequences, and GeneCompass requires both expression matrices and ranked sequences. This inconsistency complicates standardized model evaluation and hinders broader applicability across diverse downstream tasks. To evaluate the adaptability of scCompass data in training single‐cell AI models, we sampled 5 million human and 5 million mouse cells from both the scCompass and CELLxGENE. We quantified the gene numbers with detected expression values in each cell (**Figure** **5**a). For lower gene expression intervals, the cumulative frequency of CELLxGENE human data exceeded that of scCompass, suggesting that scCompass contains fewer missing values and higher data quality. This trend persisted in higher gene expression intervals, further affirming the superior data integrity of scCompass.

We pertained GeneCompass, scGPT, and Geneformer on various scales of a dataset from scCompass and CELLxGENE, and evaluated model performance in cell annotation tasks of humans and mice (Figure 5b). We also sampled 5 million human single‐cell data points from both the scCompass and Genecorpus‐30 M, respectively, to evaluate the Geneformer model (Figure S6, Supporting Information). scCompass consistently yielded higher scores, with a more pronounced advantage at larger dataset scales. We further present the cell type annotation results for the 5 million cells in scCompass with pretraining on different foundation models (GeneCompass, scGPT, and Geneformer) (Figure 5c). The results demonstrate precision and recall values consistently above 0.6, with peak values exceeding 0.8, confirming the effectiveness of our scCompass dataset across the three AI models.

We constructed scalable, AI‐ready datasets from scCompass and performed pretraining on SOTA single‐cell foundation models. Using standardized cell type annotation tasks, we conducted a unified evaluation across all models.

### 2.6 Interactive System for Single‐Cell Transcriptome Data Sharing and Analysis

**Figure 6:**
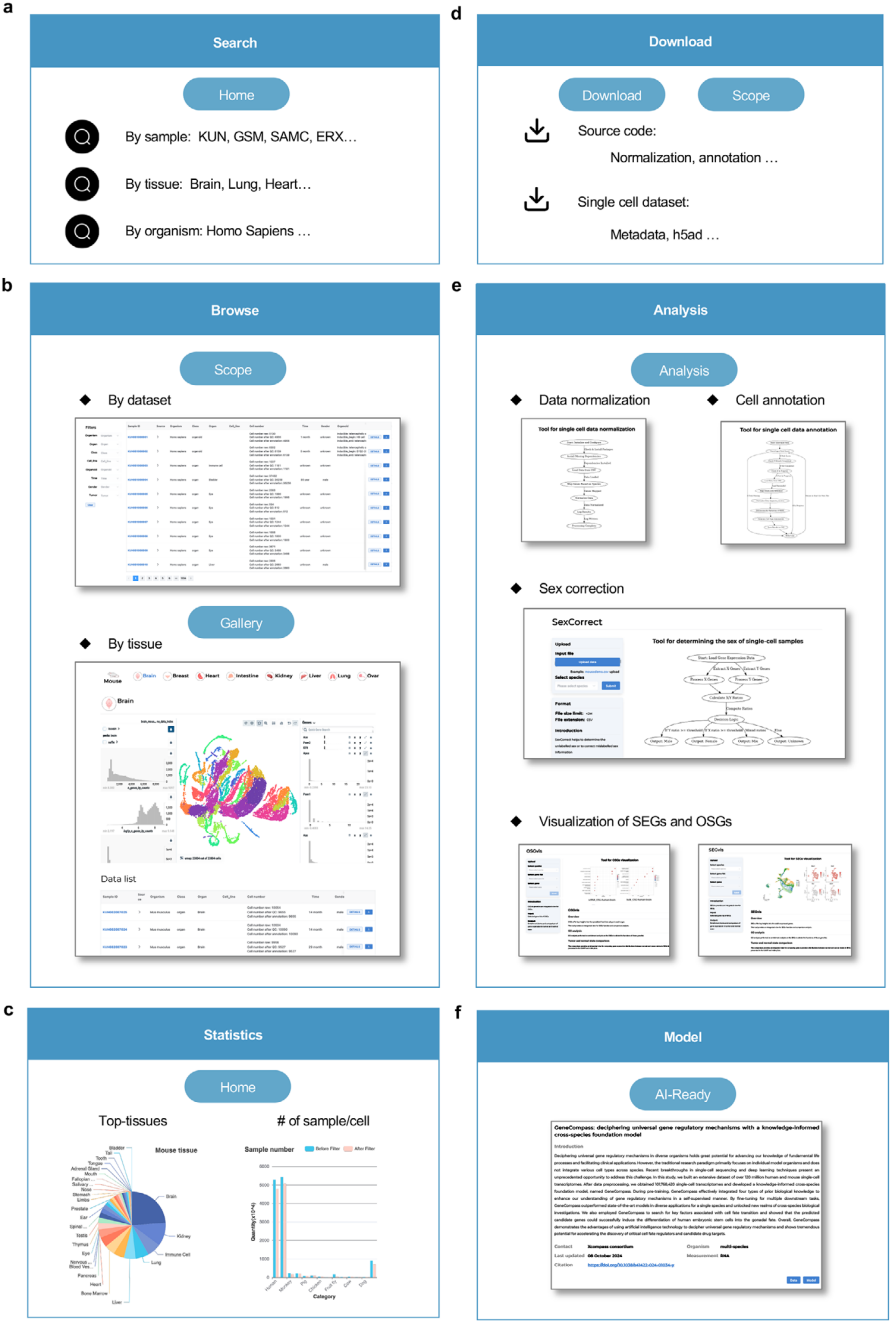
Illustration of different modules in the scCompass system. a) Search module accepts diverse search entries including sample ID, tissue, and organism. b) Interactive visualization in the Browse section for individual datasets of single‐cell sample and integrated datasets of the same tissue. c) Statistics figures show the top tissues that have the most numbers of samples or cells across all datasets. d) Download feature provides source code and h5ad file for each sample. e) The analysis section includes three online tools scNorm, cellAnno, and sexCorrect for data normalization, cell annotation, and sex determination. f) The Model module contains models and embeddings that have been trained or fine‐tuned with scCompass.

We provide a user‐friendly interface incorporating search, browse, statistics, download, analysis, and model features for scCompass exploration (http://www.bdbe.cn/kun). The system allows users to search and filter datasets (**Figure** **6**a) and provides interactive visualization of expression patterns for cells and genes in a single sample on the Scope page and as a tissue atlas on the Gallery page (Figure 6b). Statistics for the entire dataset are provided, categorized by species and organ types (Figure 6c). To enhance data accessibility, individual count matrix (h5ad format), related metadata, and analysis source code are all available for download in either the Download or Scope data tables (Figure 6d). Several online tools provide coding‐free solutions for subsequent data annotation, normalization, and extension of the scCompass dataset (Figure 6e).

- scNorm is for pre‐processing of single‐cell data. The input is a single‐cell count matrix that has column names representing official gene symbols and row names representing cell IDs.
- CellAnno processes data for cell annotation. The input is a single‐cell count matrix that has column names representing official gene symbols and row names representing cell IDs.
- SexCorrect helps to determine the unlabelled sex or to correct mislabelled sex information. A pseudo‐bulk FPKM matrix has row names that represent samples and column names that represent official gene symbols is used as input.
- OSGvis provides an integrated view of OSGs with enrichment analysis of the OSG list and tissue level comparison between tumor and normal state.
- SEGvis provides an integrated view of SEGs with enrichment analysis of the SEG list and tissue level comparison between tumor and normal state.

In the AI‐ready section, the model feature includes multi‐scale, model‐friendly datasets of varying sizes for three foundation models, as well as pre‐trained models and integrated embeddings (Figure 6f).

We developed an interactive single‐cell transcriptome sharing and analysis system based on scCompass, offering downloadable resources and model‐friendly datasets.

## 3 Discussion

We proposed scCompass, an integrated multi‐species scRNA‐seq database for AI‐ready. First, we applied a unified, standardized QC process achieving 105 million high‐quality single‐cell transcriptomes, and we corrected the sex attribute of sample metadata. Then we utilized tools to annotate cell types, constructing single‐cell atlases for each species. With this large‐scale dataset, we identified SEGs and OSGs in both humans and mice, SEGs are highly consistent in different tissues and can be used as reference genes in single‐cell resolution, and OSGs can offer valuable insights into organ functions. To facilitate the training of AI foundation models, we curated model‐compatible datasets at multiple scales and provided comprehensive benchmarking results using several SOTA pretrained models. Lastly, we developed a multi‐functional interactive data sharing and analysis system, enhancing accessibility and usability for research.

Currently, scCompass focuses exclusively on single‐cell transcriptomic data, limiting its scope to a single modality. As multimodal omics^[^ ^43^ ^]^ analysis becomes increasingly important, there is a pressing need to expand beyond transcriptomics to incorporate additional data types, such as spatial transcriptomics and epigeomics. At present, we support rapid adaptation for single‐cell foundation models. Future updates will include enhanced AI‐ready features,^[^ ^18^, ^44^ ^]^ such as data‐as‐a‐service (DaaS), model‐as‐a‐service (MaaS), and a secure, unified evaluation framework using federated learning, aimed at enabling comprehensive, integrated, and scalable data analysis.

In conclusion, scCompass is a high‐quality, shareable, AI‐ready database through unified and consistent standardization processes. It enables researchers in the life sciences and artificial intelligence fields to access and utilize the largest single‐cell database. The data sharing system provides new potential for large‐scale biological insights and establishes an essential infrastructure for the AI for Science research paradigm.

## 4 Experimental Section

### Multi‐Species Data Collection and Preprocessing

15 337 single‐cell RNA sequencing (scRNA‐seq) samples were acquired from 13 species (collected by August 2023), including human, mouse, monkey, roundworm, zebrafish, fruit fly, rat, pig, bovine, dog, horse, chicken, and sheep. These datasets were retrieved from various sources, including the Gene Expression Omnibus (GEO) of the National Center for Biotechnology Information (NCBI), European Molecular Biology Laboratory‐European Bioinformatics Institute (EMBL‐EBI) ArrayExpress, and the China National Center for Bioinformation (CNCB), using the keywords combinations such as “high throughput sequencing + 10X”, “RNA‐seq + cellranger” and “10X RNA‐seq”. After validating the integrity of the Sequence Read Archive (SRA) files, standardized tools were used, including sratoolkit^[^ ^45^ ^]^ (version 2.11.0) and fast‐dump (version 0.1.6), for data processing. Once the SRA files were converted to fastq format, the resulting fastq files were analyzed with CellRanger^[^ ^46^ ^]^ (version 7.0.1) default parameters to generate gene expression matrices with the reference genome (human: hg38, mouse: mm10, monkey: Mul_10, rat: mRatBN7.2, bovine: ARS‐UCD1.2, roundworm: WBcel235, dog: UU_Cfam_GSD_1.0, zebrafish: GRXzll, fruit fly: BDBP6.32, horse: EquCab3.0, chicken: gca000002315v5.GRCg6a, sheep: Oar_rambouillet_v1.0, pig: Sscrofall.1) for each species,^[^ ^47^ ^]^ which were then used for downstream analysis. In the database, the data which collected consists entirely of 10X Genomics data, same processing pipeline was applied, and all commands were made publicly available on GitHub (https://github.com/CNICDS/scCompass) for reproducing.

### Quality Control and Data Filtering

To ensure data quality and biological relevance, unified filtering was applied at both the sample and cell levels. First, single‐cell sequencing samples with fewer than 200 detected genes or fewer than 3 cells were excluded from further analysis. At the cellular level, cells expressing less than 7 protein‐coding or miRNA genes, and cells with a proportion of mitochondrial gene expression exceeding 15% were filtered out. Cells with an expressed gene count exceeding 3 standard deviations from the mean in each sample's gene expression matrix were also removed, and excluded genes not found in the core gene list to prioritize high‐quality data. After applying these QC measures, 15 270 samples from 13 species, containing 104637783 single‐cell datasets for further analysis were retained.

### Cell Type Annotation

Due to the large scale and high dimensionality of single‐cell data, manual cell‐type annotation was labor‐intensive and subject to human bias. Thus, for human cell type annotation, the SCimilarity^[^ ^48^ ^]^ tool was employed, while for mouse data, ScMayoMap^[^ ^23^ ^]^ was used as the reference annotation database. For the other 11 species, including zebrafish, homologous genes between each species and human, followed by cell type annotation using SCimilarity were mapped. Moreover, the low‐quality annotated cells (cell numbers per annotated cell type less than 20) were dropped.

### Sex Correction Analysis

The proportion of chrX and chrY genes was first calculated in sex‐specific organs, namely the testis and ovary, from both human and mouse samples. The list of chrX and chrY genes was downloaded from Ensembl.^[^ ^47^ ^]^ To determine sex, the Y‐Ratio and X‐Ratio were defined as follows:

(1) $$Y-\mathrm{Ratio}\;=\;\mathrm{SR}Y_{\mathrm{expr}}/\mathrm{chr}Y_{\mathrm{expr}}$$

(2) $$X-\mathrm{Ratio}\;=\frac{\mathrm{chr}X_{\mathrm{uniq}-\mathrm{expr}}}{\mathrm{chr}X_{\mathrm{expr}}}$$

Here, SRY_expr_ refers to the expression of SRY gene (SRY^[^ ^49^ ^]^ for humans, Sry^[^ ^50^ ^]^ for mice), chrY_expr_ refers to the summary expression of chrY genes. chrX_uniq‐expr_ refers to the summary expression of unique genes of chrX, excluding homologous genes between chrX and chrY, and chrX_expr_ refers to the summary expression of chrX genes.

The sex‐specific organs, testis, and ovary were utilized to calculate Y‐Ratio and X‐Ratio values, establishing an initial threshold for sex determination. Using these initial thresholds, sex predictions across different samples were performed and compared with existing sex labels. The thresholds based on incorrectly predicted samples were then refined, optimizing the final criteria for sex determination.

A human individual was determined as male if the Y‐Ratio is ≥0.001188 or the X‐Ratio ≤ 0.000070 with a Y‐Ratio > 0.000106. Female classification requires an X‐Ratio ≥ 0.000001 and Y‐Ratio ≤ 0.000106. An X‐Ratio > 0.000070 with a Y‐Ratio between 0.001188 and 0.000106 indicates a “mixed” chromosomal profile. Samples that did not meet these criteria were labeled as “unknown”.

A mouse individual was determined as male if the Y‐Ratio was ≥0.000065 or the X‐Ratio ≤ 0.000008 with a Y‐Ratio between 0.000004 and 0.000065. Female classification requires an X‐Ratio ≥ 0.000555 and Y‐Ratio between 0.000004 and 0.000065, or X‐Ratio > 0 with Y‐Ratio ≤ 0.000004. An X‐Ratio between 0.000008 and 0.000555 with a Y‐Ratio between 0.000004 and 0.000065 indicates a “mix” chromosomal profile. Samples that did not meet these criteria were labeled as “unknown”.

### SEGs Identification and Assessment

The proportion of zero counts was first quantified for each gene across all cells in human and mouse samples by calculating the ratio of cells with zero counts to the total number of cells. Genes were ranked by their zero‐rate values. Next, pseudo‐bulk processing was performed on single‐cell data and generated FPKM^[^ ^51^, ^52^ ^]^ (fragments per kilobase of transcript per million mapped reads) matrix from different species and calculated the CV^[^ ^53^, ^54^, ^55^ ^]^ for each gene as the ratio of the standard deviation to the mean expression. Genes were then ranked based on their CVs. The rankings from the zero rate and CV were combined to generate a final ranking of gene stability.

To assess gene stability, HKGs as a reference we used.^[^ ^56^ ^]^ Same number of HKGs was selected from top‐ranked stable genes, defining these as SEGs. To evaluate the expression stability of these gene sets across different cell types and biological systems, single‐cell data was pseudo‐bulk processed from ten organs (brain, lung, intestine, kidney, bone marrow, liver, skin, heart, breast, and spleen) and converted the data to FPKM. The expression patterns of SEGs and HKGs were visualized using heatmaps generated with the ComplexHeatmap^[^ ^57^ ^]^ R package.

Clustering was performed on each single‐cell RNA sequencing dataset using the Seurat^[^ ^58^ ^]^ package, and clustering performance was evaluated using the Calinski‐Harabasz index^[^ ^27^ ^]^ and Silhouette score.^[^ ^28^ ^]^ Three scenarios: (i) using all expressed genes, (ii) using HKGs from bulk RNA‐seq data, and (iii) using SEGs identified in this study were compared. Clustering was repeated 20 times with random subsampling to account for variability. The SEGs and HKGs in human and mice were replaced with their respective homologous genes, yielding a set of common SEGs and common HKGs. To characterize the evolutionary conservation of those common genes, phyloP^[^ ^59^, ^60^ ^]^ scores were downloaded for the human (hg38) and mouse (mm10) genomes from the UCSC Genome Browser.^[^ ^61^ ^]^ Exonic bases were mapped based on GENCODE gene annotations, and a mean conservation score was calculated for each gene. The correlation between gene conservation was assessed using Pearson correlation coefficients^[^ ^62^ ^]^ and these features between common SEGs and common HKGs in both humans and mice.

The overrepresentation of common SEGs and common HKGs was evaluated by comparing each gene set to GO^[^ ^63^ ^]^ terms using the GO database with R package clusterProfiler.^[^ ^64^ ^]^ Statistical significance was assessed using Fisher's exact test, and the top enriched GO terms were merged for interpretation.

### OSGs Definition and Analysis

For OSGs identification, the zero rate of genes in single‐cell data was calculated from different organs in both human and mouse datasets. Organ‐specific genes were defined as the proportion of zero values was no more than 90% of cells in a given organ but expressed in no more than two organs.

To enhance gene number and expression correlation in high‐throughput single‐cell mRNA datasets, data was pooled from 100 cells within the same cluster, and generate pseudo‐cells to support genetic network analysis.^[^ ^65^ ^]^

Based on these pseudo‐cell datasets, the tool pySCENIC^[^ ^66^ ^]^ was used to infer regulatory circuits and identify transcription factor‐target gene (TF‐TG) interactions for organ‐specific genes. Only TF‐TG interactions with the highest regulatory impact were retained for constructing regulatory networks (30 weights). These representative TF‐TG networks were visualized using Cytoscape^[^ ^67^ ^]^ and the R package igraph.^[^ ^68^ ^]^ Finally, GO enrichment analysis was performed on the organ‐specific genes to uncover their functional roles within each organ.

### Evaluation of the Dataset Quality for AI‐Ready

To assess the quality of our dataset, 5 million cells each from GeneCompass and CELLxGENE for both humans and mice were randomly selected. The gene numbers with non‐zero expression values in each cell were initially quantified, then binned these counts in 1000‐gene intervals. To validate the quality of our constructed datasets, the sampled data was used to build pre‐training datasets and trained them on the specific foundational models, GeneCompass,^[^ ^8^ ^]^ scGPT,^[^ ^9^ ^]^ and Geneformer.^[^ ^10^ ^]^ The models’ performance was then evaluated through downstream tasks involving cell type annotation. Initially, datasets of different data sizes 100 thousand, 200 thousand, 500 thousand, 1 million, 2 million, and 5 million were created. During the pre‐training phase, It was to the hyperparameters specified in the original papers of these models, ensuring consistency in experimental setup.

To ensure a fair comparison, the pre‐training process for each model involved keeping the hyperparameter settings consistent with those outlined in their respective publications, such as learning rate, batch size, and optimizer. The number of epochs based on the dataset size was adjusted, setting them at 3 for the datasets of 100 thousand, 200 thousand, 500 thousand, 1 million, 2 million, and 5 million cells, respectively. To further evaluate the performance of the trained models, the human multiple sclerosis (hMS),^[^ ^8^ ^]^ lung (hLung),^[^ ^69^ ^]^ and liver (hLiver)^[^ ^70^ ^]^ datasets were used, as well as mouse brain (mBrain),^[^ ^71^ ^]^ lung (mLung)^[^ ^72^ ^]^ and pancreas (mPancreas)^[^ ^73^ ^]^ datasets for downstream cell annotation tasks. The hyperparameter settings for each model in these downstream tasks were as follows: for GeneCompass, the learning rate was set to 5e‐5, batch size to 16, and the number of training epochs to 50; for Geneformer, the learning rate was set to 5e‐5, batch size to 16, and epochs to 50; and for scGPT, the learning rate was similarly set to 5e‐5, batch size to 16, and epochs to 50.

To evaluate the performance for Geneformer with original dataset and scCompass. Single‐cell data of varying scales from scCompass and Genecorpus‐30 m was randomly sampled to build pre‐training datasets and trained them on Geneformer. The number of epochs based on the dataset size was adjusted, setting them at 10, 8, 7, 5, 4, and 3 for the datasets of 100 thousand, 200 thousand, 500 thousand, 1 million, 2 million, and 5 million cells, respectively.

Accuracy, recall, and precision were employed as evaluation metrics to assess the performance across the three datasets. Accuracy quantifies the proportion of all instances (both positive and negative) that were correctly classified by the model, providing an overall measure of correctness. The accuracy (A) is defined as:

(3) $$A=\frac{TP+TN}{TP+\mathrm{TN}+\mathrm{FP}+FN}$$

where TP represents True Positives, which are the number of correctly classified positive instances, and TN represents True Negatives, the number of correctly classified negative instances. FP represents False Positives, the number of negative instances incorrectly classified as positive, and FN represents False Negatives, which are the number of positive instances incorrectly classified as negative.

Recall, also known as sensitivity, quantifies the proportion of true positive instances that are correctly identified by the model, providing a measure of completeness. The recall (R)^[^ ^74^ ^]^ is defined as:

(4) $$R=\frac{TP}{TP+FN}$$

Precision quantifies the proportion of correctly identified positive predictions among all instances predicted as positive, reflecting the accuracy of positive predictions. The precision (P)^[^ ^74^ ^]^ is given by:

(5) $$P=\frac{TP}{TP+FP}$$

Accuracy is a useful metric when the dataset is balanced, as it reflects the model's overall performance across all classes. Furthermore, these metrics provided a comprehensive understanding of each model's performance in terms of both identifying true positives effectively (Recall) and minimizing false positives (Precision). The achieved models’ performance was evaluated using two cell‐type annotation tasks, which included three human datasets (hLiver, hLung, hMs) and three mouse datasets (mBrain, mLung, mPancreas).

#### Database Development

The scCompass database used PostgreSQL (https://www.postgresql.org) to index and store the metadata. FastAPI (https://fastapi.tiangolo.com) was used to develop the interface to query the database or files. Nginx (https://nginx.org/) and Gunicorn WSGI HTTP server (https://docs.gunicorn.org/en/stable/) were used to serve the application.

The database used the Vue3.0 framework (https://vuejs.org) in conjunction with ElementPlus UI (https://element‐plus.org/), Vue Router (https://router.vuejs.org/zh/) and Vite tool (https://vitejs.dev) for front end service. Interactive visualization diagrams were created with the libraries (https://github.com/Novartis/cellxgene‐gateway, https://echarts.apache.org/).

### Appendix

#### The X‐Compass Consortium Members

##### Institute of Zoology, Chinese Academy of Sciences

Xin Li, Hongmei Wang, Baoyang Hu, Wei Li, Fei Gao, Jingtao Guo, Leqian Yu, Qi Gu, Weiwei Zhai, Zhengting Zou, Guihai Feng, Wenhao Liu, Yao Tian, Chen Fang, Jingxi Dong, Yana Liu, Jingqi Yu, Wenhui Wu, Stella Lin, Cong Li, Yu Zou, Yongshun Ren, Fan Li, Yixiao Zhao, Yike Xin, Longfei Han, Shuyang Jiang, Kai Ma, Qicheng Chen, Haoyuan Wang, Huanhuan Wu, Chaofan He, Yilong Hu, Shuyu Guo, Yiyun Li

#### Computer Network Information Center, Chinese Academy of Sciences

Yuanchun Zhou, Yangang Wang, Xuezhi Wang, Pengfei Wang, Fei Li, Zhen Meng, Zheng Li, Zaitian Wang, Ping Xu, Wentao Cui, Zhilong Hu, Yang Wang, Huimin He, Shan Zong, Jiajia Wang, Yan Chen, Chunyang Zhang, Chengrui Wang, Qingqing Long, Ran Zhang, Meng Xiao, Qinmeng Yang, Zijian Wang, Yining Wang

#### Institute of Computing Technology, Chinese Academy of Sciences

Yiqiang Chen, Yi Zhao, Xiaodong Yang, Dechao Bu, Xin Qin, Jiaxin Qin, Zhaohui Yang, Chenhao Li, Zhufeng Xu, Zeyuan Zhang, Xiaoning Qi, Shubai Chen, Wuliang Huang, Yaning Li

#### Institute of Automation, Chinese Academy of Sciences

Ge Yang, Jing Liu, Guole Liu, Jie Jiang, Xingjian He, Liqun Zhong, Yaoru Luo, Jiaheng Zhou, Zichen Wang, Qinxuan Luo, Ziwen Liu, Ao Li, Teng Wang, Yiming Huang, Handong Li

#### Academy of Mathematics and Systems Science, Chinese Academy of Sciences

Yong Wang, Shihua Zhang, Jiahao Zhang, Yiyang Zhang, Shirui Li, Zhongming Liang, Zhenpeng Man, Kangning Dong, Qunlun Shen

## Conflict of Interest

The authors declare no conflict of interest.

## Author Contributions

P.W., W.L., J.W., Y.L., and P.L. contributed equally to this work. Y.Z., X.L., G.F., X.W., Z.M., and S.Z. supervised the project; Y.Z., X.L., G.F., X.W., Z.M., P.W., and J.W. conceived and designed the study; P.W., W.L., J.W., Y.L., P.L., P.U, W.C., R.Z., Q.J.D., C.Z., C.W., G.L., H.J. involved in collecting and preprocessing scRNA‐seq data, designing the algorithm and performed the experiments; P.W., W.L., J.W., and Y.C. designed the interactive system; P.W., W.L., J.W., Y.L., and P.L. wrote the manuscript; Z.H., C.F., H.X., Y.Z., M.X., and S.C. provided assistance in data collection and analysis. The X‐COMPASS Project Consortium members collaborated in the paper discussions. All authors read and approved the final version of the manuscript.

## Supporting information



Supporting Information

## Data Availability

The data that support the findings of this study are openly available in scCompass at https://www.bdbe.cn/kun/#/home.
