# Supplementary material for: scCompass: An Integrated Multi‐Species scRNA‐seq Database for AI‐Ready
Source: Adv Sci (Weinh). 2025 May 2;12(25):2500870. doi: 10.1002/advs.202500870 (PMC12224968; doi:10.1002/advs.202500870)
Supplement: Supplementary file 1 — Supporting Information [file ADVS-12-2500870-s001.docx]

**Supporting Information**

**Fig. S1| scCompass Statistical Analysis. a,** Statistics of pre- and post-quality control results for datasets, samples, tissues, and cell counts across thirteen species in scCompass. **b,** Proportion of tumor samples across the thirteen species. **c,e,** Proportion distribution of X and Y chromosomes in human and mouse tesits, with X (blue) and Y (orange). **d,f,** Proportion distribution of X and Y chromosomes in human and mouse ovaries, with X (blue) and Y (orange).


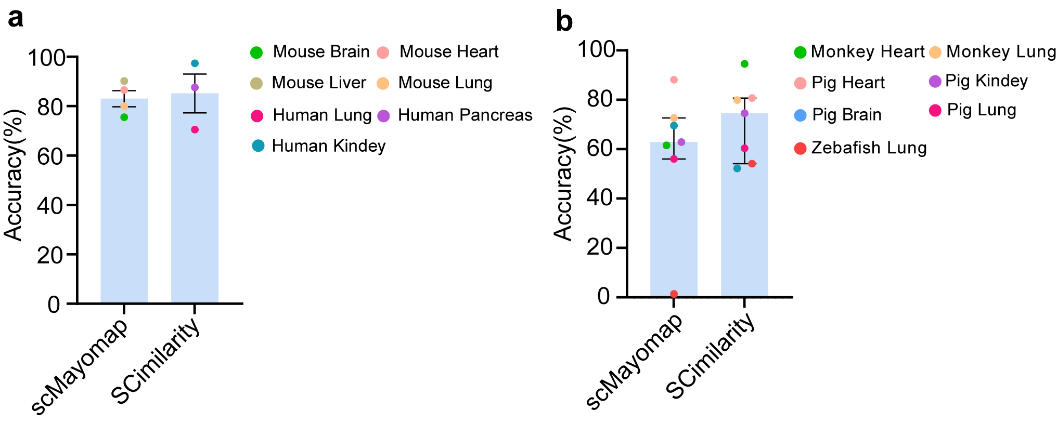


**Fig.S2| Accuracy of cell type annotation across different species and tissues.** **a**, Accuracy of cell type annotation in mouse and human tissues using scMayomap and Sciminarity tools. Tissues include mouse brain^77^, mouse heart^78^, mouse liver^79^, mouse lung^78^, human lung^80^, human pancreas^81^, and human kidney^82^. **b**, Accuracy of cell type annotation in monkey, pig, and zebrafish tissues using scMayomap and Sciminarity tools. Tissues include monkey heart^83^, monkey lung^83^, pig heart^84^, pig kidney^84^, pig brain^84^, pig lung^84^ and zebrafish lung^85^.

**Fig. S3| Single-cell Atlas Constrution of 11 species.** tSNE plot showing single-cell atlas of Zebrafish, Fruit fly, Rat, Pig, Chicken, Bovine, Roundworm, Dog, Sheep, and Horse, dots with colors represent different tissues.


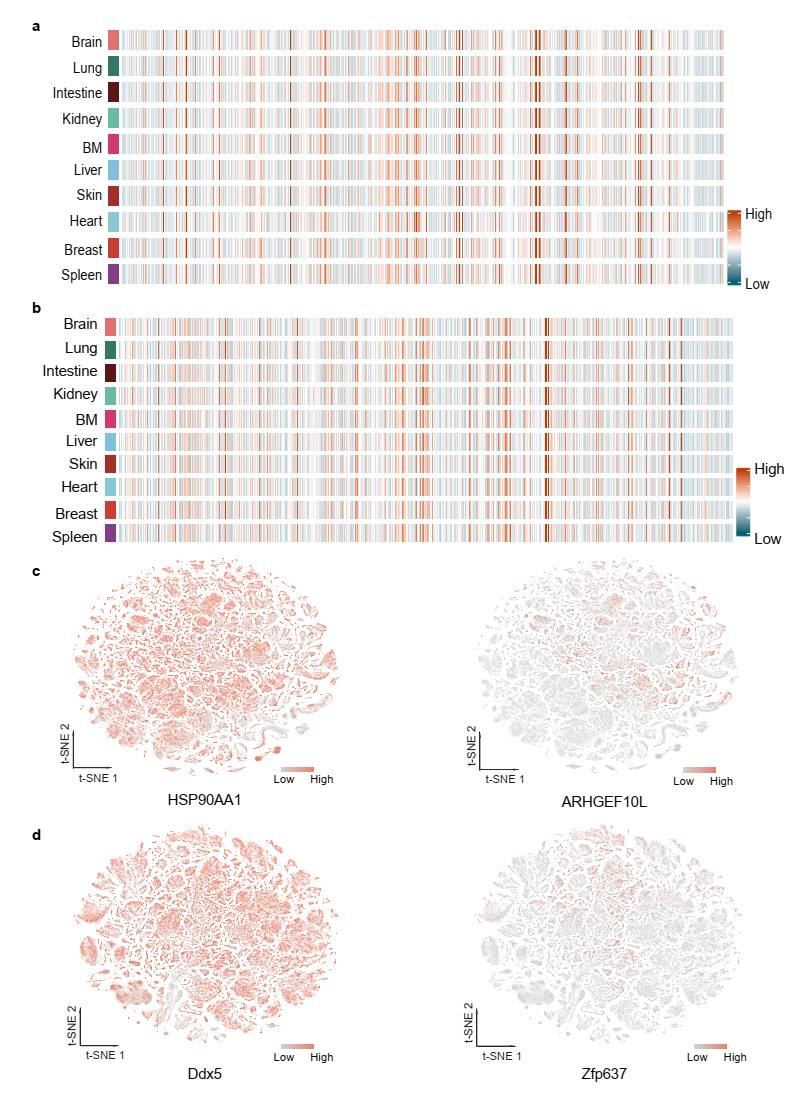


**Fig. S4| SEG analysis of human and mouse**. Heatmap showing the distribution of HKGs in the top 10 organs for human (**a**) and mouse (**b**). **c.** tSNE visualization showing the expression pattern of HSP90AA1 of unique hSEGs and ARHGEF10L of unique hHKGs, respectively, in indicated the top30 organs of human. **d,** tSNE visualization showing the expression pattern of Ddx5 of unique mSEGs and Zfp637 of unique mHKGs, respectively, in indicated the top30 organs of mouse.


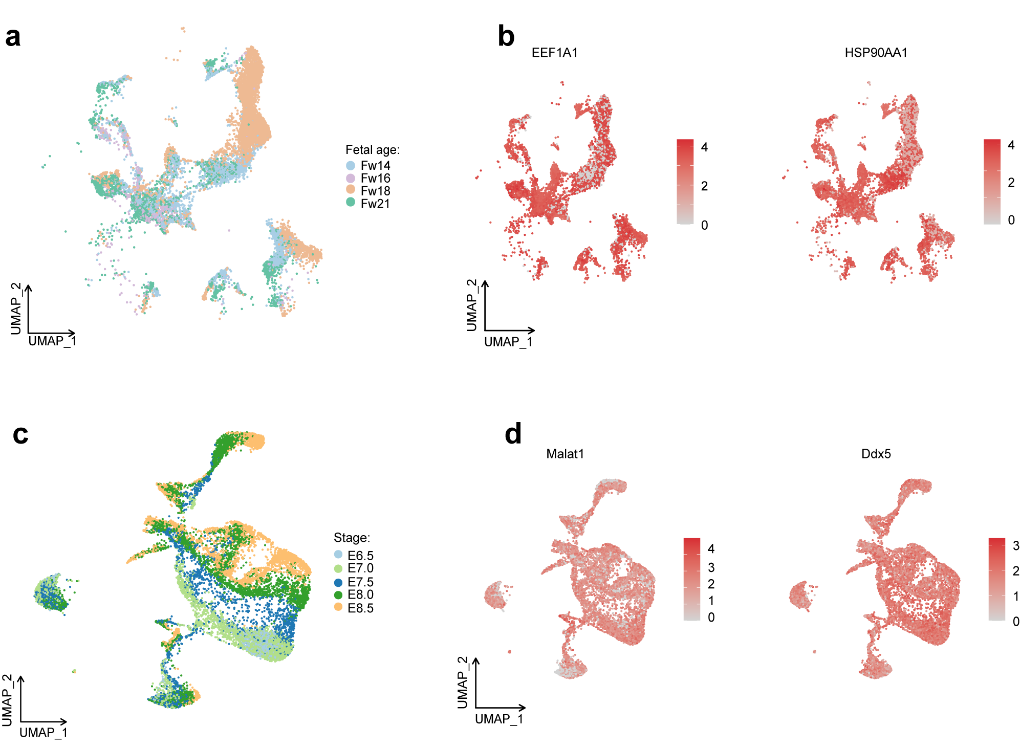


**Fig.S5| Expression patterns of SEGs in time-series data.a**,Single-cell UMAP dimensionality reduction results of human fetal liver at different developmental stages, including four time points: Fw14(fetal 14 weeks), Fw16(fetal 16 weeks), Fw18(fetal 18 weeks), and Fw21(fetal 21 weeks). **b**, Expression patterns of EEF1A1 and HSP90AA1 genes in human fetal liver at different developmental stages. **c**, Single-cell UMAP dimensionality reduction results of mouse embryos at different developmental stages, covering six time points: E6.5, E7.0, E7.5, E8.0, and E8.5. **d**, Expression patterns of Malat1 and Ddx5 genes in mouse embryos at different developmental stages.

**Fig. S6| Evaluation of scCompass AI-Ready adaptability,** Precision, Recall, Accuracy, and Macro F1 of Geneformer models trained on various scales of single-cell samples, evaluated on hMS and hLiver for cell type annotation. The green dots line represents pretraining with scCompass data, while blue dots line represents pretraining with Geneformer data.

**References**

77. Allen WE, Blosser TR, Sullivan ZA, Dulac C, Zhuang X. Molecular and spatial signatures of mouse brain aging at single-cell resolution. *Cell* **186**, 194-208 e118 (2023).

78. Tabula Muris C. A single-cell transcriptomic atlas characterizes ageing tissues in the mouse. *Nature* **583**, 590-595 (2020).

79. Palovics R*, et al.* Molecular hallmarks of heterochronic parabiosis at single-cell resolution. *Nature* **603**, 309-314 (2022).

80. He P*, et al.* A human fetal lung cell atlas uncovers proximal-distal gradients of differentiation and key regulators of epithelial fates. *Cell* **185**, 4841-4860 e4825 (2022).

81. Wang G*, et al.* Integrating genetics with single-cell multiomic measurements across disease states identifies mechanisms of beta cell dysfunction in type 2 diabetes. *Nat Genet* **55**, 984-994 (2023).

82. Young MD*, et al.* Single cell derived mRNA signals across human kidney tumors. *Nat Commun* **12**, 3896 (2021).

83. Han L*, et al.* Cell transcriptomic atlas of the non-human primate Macaca fascicularis. *Nature* **604**, 723-731 (2022).

84. Wang F*, et al.* Endothelial cell heterogeneity and microglia regulons revealed by a pig cell landscape at single-cell level. *Nat Commun* **13**, 3620 (2022).

85. Wang R*, et al.* Construction of a cross-species cell landscape at single-cell level. *Nucleic Acids Res* **51**, 501-516 (2023).
